# Strigolactones Stimulate Arbuscular Mycorrhizal Fungi by Activating Mitochondria

**DOI:** 10.1371/journal.pbio.0040226

**Published:** 2006-06-27

**Authors:** Arnaud Besserer, Virginie Puech-Pagès, Patrick Kiefer, Victoria Gomez-Roldan, Alain Jauneau, Sébastien Roy, Jean-Charles Portais, Christophe Roux, Guillaume Bécard, Nathalie Séjalon-Delmas

**Affiliations:** **1**UMR 5546, Pôle de Biotechnologies Végétales, Castanet-Tolosan, France; **2**Laboratoire Biotechnologie-Bioprocédés, L'Institut National des Sciences Appliquées, Toulouse, France; **3**IFR40, Pôle de Biotechnologies Végétales, Castanet-Tolosan, France; **4**UMR 5546, Centre National de la Recherche Scientifique–Université Toulouse III, Pôle de Biotechnologies Végétales, Castanet-Tolosan, France; The Salk Institute for Biological StudiesUnited States of America

## Abstract

The association of arbuscular mycorrhizal (AM) fungi with plant roots is the oldest and ecologically most important symbiotic relationship between higher plants and microorganisms, yet the mechanism by which these fungi detect the presence of a plant host is poorly understood. Previous studies have shown that roots secrete a branching factor (BF) that strongly stimulates branching of hyphae during germination of the spores of AM fungi. In the BF of *Lotus,* a strigolactone was found to be the active molecule. Strigolactones are known as germination stimulants of the parasitic plants *Striga* and *Orobanche*. In this paper, we show that the BF of a monocotyledonous plant, *Sorghum,* also contains a strigolactone. Strigolactones strongly and rapidly stimulated cell proliferation of the AM fungus Gigaspora rosea at concentrations as low as 10 ^−13^ M. This effect was not found with other sesquiterperne lactones known as germination stimulants of parasitic weeds. Within 1 h of treatment, the density of mitochondria in the fungal cells increased, and their shape and movement changed dramatically. Strigolactones stimulated spore germination of two other phylogenetically distant AM fungi, Glomus intraradices and *Gl. claroideum*. This was also associated with a rapid increase of mitochondrial density and respiration as shown with *Gl. intraradices*. We conclude that strigolactones are important rhizospheric plant signals involved in stimulating both the pre-symbiotic growth of AM fungi and the germination of parasitic plants.

## Abstract 2

Strigolactones from *Sorghum* promote growth of multiple arbuscular mycorrhizal fungi, underscoring the importance of these molecules in the symbiotic relationship between plant roots and the rhizosphere.

## Introduction

In the immediate area surrounding plant roots, called the rhizosphere, roots are in active contact with soil-borne microorganisms. Besides the beneficial or competitive interactions between roots and microorganisms that involve microbial nutrient cycling, plant supply of carbon substrates, and competition for nutrient sources, plant roots and soil microbes also interact more intimately. Roots exude not only large quantities of amino acids, organic acids, sugars, vitamins, purines, nucleosides, enzymes, hormones, inorganic ions, and CO _2_, but also small amounts of a variety of flavonoids, terpenoids, and other secondary metabolites [ 1] that serve as early chemical mediators of more specific plant-microbe interactions [ 2]. Some of them act as chemoattractants [ 3, 4], as antibiotics that confer resistance to bacterial pathogens [ 5], or as root signals that specifically induce microbial genes necessary for the establishment of compatible associations with plants [ 6].

About 80% of all plant species form intimate symbiotic associations with a class of ubiquitous soil microorganisms called arbuscular mycorrhizal (AM) fungi, yet little is known about how these fungi detect the presence of their plant hosts. As obligate biotrophs, the survival of these fungi depends on their ability to associate rapidly with plant roots and to set up symbiotic relationships with them. Spores of AM fungi can germinate in the absence of a host (non-symbiotic growth); however, further growth and branching of germinating hyphae prior to root infection (pre-symbiotic growth) require the presence of compounds released by roots [ 7]. Among the diversity of molecules present in root exudates and active in hyphal branching, flavonoids have often been proposed to switch AM fungi from non- to pre-symbiotic growth [ 8, 9]; micromolar concentrations of some of them can stimulate growth of AM fungi. However, the roots of chalcone synthase mutants of maize, which are deficient in flavonoid synthesis, are colonised normally by AM fungi, demonstrating that flavonoids are not essential for the process [ 10].

In earlier studies, a branching factor (BF) from hairy root cultures of Daucus carota was discovered that strongly stimulates branching of the germinating hyphae of Gigaspora spp. [ 11, 12]—a response similar to that typically observed when hyphae of AM fungi grow in the presence of living roots. BF induces mitosis in the hyphae [ 12], which is necessary to form new hyphal branches and increase root–fungus contacts. We found this BF also in several other mycotrophic plants [ 12]. BF first induces expression of genes related to mitochondrial activity in the fungus (after 1h), then increases the rate of respiration (after 1h 30 min) and mitochondrial reorganization (after 4h) before stimulating fungal ramification (after 24h) [ 13], suggesting that the branching response is the result of a metabolic switch.

Preliminary investigations of BF of D. carota indicated that the active component is a low-molecular-weight lipophilic molecule present in root exudates at extremely low concentration [ 12]. Among candidate molecules in root exudates that share these characteristics and are already known to be involved in plant–soil organism interactions are the strigolactones—potent germination stimulants of parasitic weeds such as *Striga* and *Orobanche* [ 14] ( Figure S1). Like AM fungi, these parasitic plants are obligate biotrophs whose survival depends on rigorous host recognition [ 15]. Recently Akiyama et al. [ 16] demonstrated that the BF of the dicotyledenous (dicot) plant Lotus japonicus contains the strigolactone 5-deoxy-strigol. This report also showed that 5-deoxy-strigol, strigol, sorgolactone, and the strigolactone analogue GR24 stimulate hyphal branching of the AM fungus *Gi. margarita* at subnanomolar concentrations.

To substantiate the hypothesis of strigolactones as important stimulants of AM fungi, our aim was to examine whether: i) the BF isolated from a monocotyledonous (monocot) plant species contains strigolactones; ii) among known stimulants of *Striga* and *Orobanche* seed germination, strigolactones specifically stimulate AM fungi [ 15]; iii) strigolactones not only stimulate hyphal branching of germinating spores of AM fungi but also are the molecules responsible for activating mitochondria, the typical response to BF [ 13]; iv) strigolactones are active on more than one AM fungal species.

Our data show that strigolactones must indeed be a widespread class of rhizospheric stimulants of AM fungi produced by dicots as well as monocots. They also demonstrate that these molecules provoke a rapid and strong cellular response targeted on mitochondrial apparatus in the fungus.

## Results

### The BF of D. carota Hairy Roots Stimulates not only AM Fungi but also Seed Germination of *Orobanche*

We first tested whether BF isolated from D. carota (carrot) hairy roots, which stimulates branching of the hyphae of AM fungi, would also stimulate germination of the seeds of the parasitic weed *Orobanche*. The percentage of germinating seeds was 6.2- to 8.9-fold higher for BF-treated seeds compared to controls. A positive control with GR24 (10 ^−7^ M), a strigolactone analogue that induces germination of parasitic weeds, increased germination an average of 6.3-fold compared to the control, showing that, in our experimental conditions, the carrot BF and GR24 had similar stimulatory activity on *Orobanche* seed germination.

### Seed Germination Stimulants of Parasitic Weeds other than Strigolactone Have No Effect on Hyphal Branching of Gigaspora rosea

Several molecules have been identified as potent stimulants of *Striga* and *Orobanche* germination: strigolactones [ 17] (from which the synthetic analogue GR24 is derived [ 18]), sesquiterpene lactones such as parthenolide and artemisinin [ 19], and dihydrosorgoleone [ 20] ( Figure S1). To see whether AM fungi respond to one or more of these compounds, we tested the effect of GR24, parthenolide, artemisinin, and dihydrosorgoleone on hyphal branching of *Gi. rosea*. We used the very sensitive bioassay of branching described by Buée et al. [ 12]. Parthenolide, artemisinin, GR24, and dihydrosorgoleone were tested at concentrations known to activate parasitic weed germination; GR24 was the only compound that gave a positive response ( Table 1).

**Table 1:**
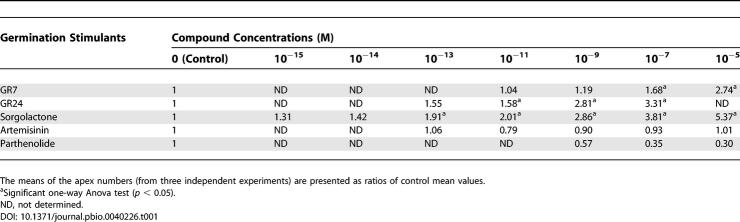
Quantitative Hyphal Branching Response of *Gi. rosea* Stimulated by Various Concentrations of Germination Stimulants of *Striga* and *Orobanche*

### *Sorghum* BF Contains the Strigolactone Sorgolactone

**Figure 1:**
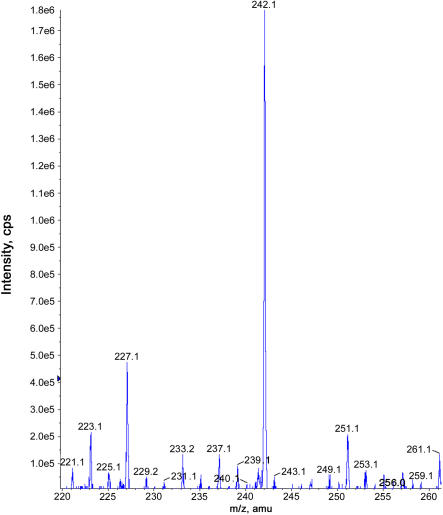
ESI-MS/MS Analysis, in the Positive EPI Scan Mode, of a Purified *Sorghum* Extract Active in a *Gi. rosea* Hyphal Branching Assay The parent ion m/z = 339 shows the characteristic daughter ion of sorgolactone [M + Na] ^+^ at m/z = 242.

5-deoxy-strigol was found to be the active substance in the BF of *L. japonicus* [ 16]. For generalisation purposes, it is essential to determine whether the BF of other plants, particularly in divergent ones like monocots, also contains strigolactones. Therefore, we analysed the BF of *Sorghum* because the strigolactones of this plant species have already been characterized *. Sorghum* contains at least two strigolactones: sorgolactone and, in smaller amounts, strigol [ 21]. We collected root exudates from *Sorghum* seedlings that had germinated for 5–30 d. The organic content of the exudates was extracted in ethyl acetate and separated using C18 fractionation. All fractions were tested for activity in the fungal branching assay. 24 h after its application, the most hydrophobic fraction (fraction 6) could be seen to stimulate hyphal branching of *Gi. rosea*. Sorgolactone was identified in this fraction by using ESI-MS/MS from the (M + Na ^+^) 339 m/z ion showing the loss of the specific D ring of strigolactones (daughter ion at 242 m/z) ( Figure 1). Fraction 6 was further separated using C18 high-performance liquid chromatography (HPLC), and the subfraction that eluted in the same position as a synthetic sorgolactone marker was collected. This fraction also stimulated hyphal branching.

The same protocol was applied to crushed roots and leaves from *Sorghum* seedlings grown for 4 wk. Sorgolactone was detected in the same C18 column fraction 6 as in the separation of root exudates (100% acetonitrile; unpublished data). This rapid method for detecting strigolactones directly in plant tissue extracts has not previously been reported in the literature and may provide an important tool for exploring their occurrence in the plant kingdom.

### Strigolactones Act on *Gi. rosea* at Very Low Concentrations

We carried out a quantitative dose-response analysis of the effect of GR24, GR7 (another strigolactone analogue that contains only the BCD rings; Figure S1), and synthetic sorgolactone on *Gi. rosea* hyphal branching ( Table 1). GR7 stimulated branching at concentrations above 10 ^−7^ M, whereas GR24 and sorgolactone were still significantly active at 10 ^−11^ M and 10 ^−13^ M, respectively. This shows that GR24 and sorgolactone are more potent branching stimulants than GR7, and thus that the A ring, absent from the GR7 molecule, is probably required for a maximum activity.

**Figure 2:**
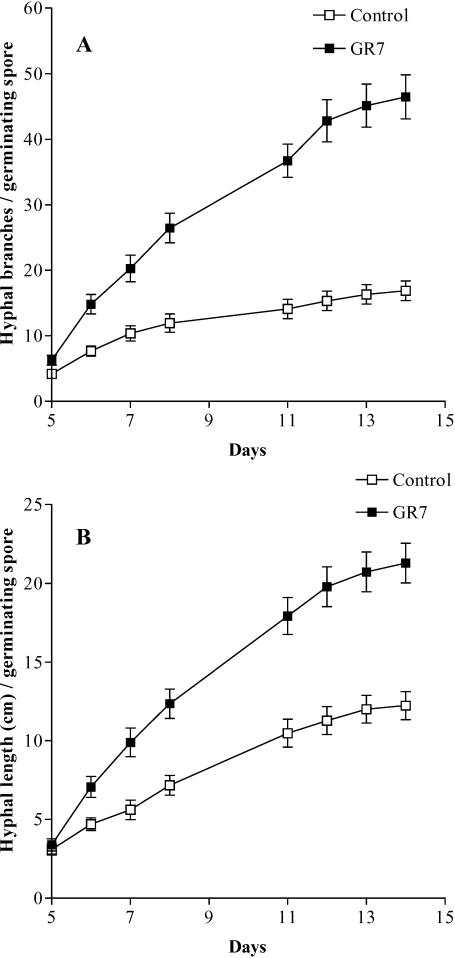
Time-Course Analyses of *Gi. rosea* Hyphal Branching (A) and Growth (B) Treated with 10 ^−7^ M GR7 in Methanol:Water (v/v) or with Methanol:Water (v/v) (Control) Number of hyphal branches and total hyphal length (including hyphal branches) were recorded and cumulated from day 5 to 14. Twenty spores were analysed per treatment. This experiment was repeated three times with similar results. Error bars show SEM.

To determine whether the stimulation of hyphal branching by strigolactone observed after 24 h was also significant after several days, hyphal branching and hyphal length were measured up to 2 wk after stimulation with 10 ^−7^ M GR7 ( Figure 2). GR7 was used for this assay because a large amount was needed and it was available commercially. Stimulation of hyphal branching by GR7 was maintained throughout the 2-wk period. At the end of the experiment, the treated spores had produced 2.8-fold more hyphal branches than control spores. The hyphae that grew from the treated spores were also longer. Hyphal branching, therefore, seems to be a durable effect, suggesting that the stimulated fungus enters a new morphogenetic program, preceding the symbiotic stage.

### The Strigolactone Analogue GR24 Induces a Rapid Mitochondrial Response in the Fungus

**Figure 3:**
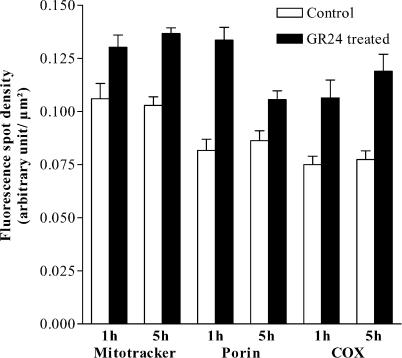
Cellular Response of Germinating Spores of *Gi. rosea* to GR24 after 1 h or 5 h of Treatment with GR24 Hyphae were stained with MitoTracker Green and treated with 0.001% acetone (control) or 30 nM GR24. Mitochondrial density is significantly higher in treated samples than in control at 1 h and 5 h ( *p* < 0.001, *n* = 26; and *p* < 0.001, *n* = 42, respectively). For porin and COX immunolabelling, hyphae were treated with acetone or GR24 (as above) and incubated with antibodies as described in Materials and Methods. The fluorescence density of porin and COX immunolabelling is significantly higher in treated hyphae than in controls both at 1 h ( *p* < 0.001, *n* = 52; and *p* < 0.05, *n* = 60, respectively) and at 5 h ( *p* < 0.05, *n* = 28; and *p* < 0.001, *n* = 29, respectively). Error bars show SEM.

Tamasloukht et al. [ 13] showed that before *Gi. rosea* cells began to branch in response to BF isolated from carrot hairy roots, they produced more mitochondria and their respiratory rate increased. These important fungal responses involve the oxidative catabolism of lipids—the main form of carbon storage in AM fungi—and the production of ATP. This raises the possibility that strigolactones in BF are responsible for stimulating fungal respiration and lipid catabolism. To address this question, we tested the effect of GR24 on mitochondrial density, shape, and movement in *Gi. rosea*. Living hyphae were stained with Mitotracker Green, a fluorescent vital stain specific for mitochondria [ 22], and observed by fluorescence microscopy [ 13]. After only 1 h of GR24 treatment, the mitochondrial density increased by 23% ( Figure 3). This phenomenon was more pronounced after 5 h of treatment (32%; Figure 3). To substantiate these data, we used monoclonal antibodies against the yeast mitochondrial cytochrome oxidase (COX) subunit I and against the yeast mitochondrial porin protein to observe the mitochondria by immunofluorescence microscopy in fixed fungal hyphae ( Figure S2). Double-labelling with the two antibodies showed clearly co-localised staining ( Figure S3). We calculated the fluorescence intensity of fungal cells labelled with each antibody before and after exposure of the fungus to GR24. Consistent with the Mitotracker Green test, we found a significant increase in mitochondrial density with the anti-porin antibody as well as with the anti-COX antibody ( Figure 3).

**Figure 4:**
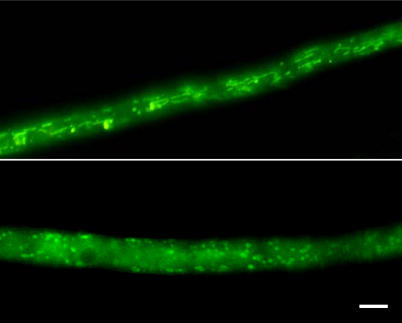
Effect of GR24 on Mitochondrial Shape and Density in Hyphae of *Gi. rosea* Stained with Mitotracker Green Hyphae were treated with 30 nM GR24 (up) or with 0.001% acetone (bottom) for 1 h. Bar = 10 μm.

**Figure 5:**
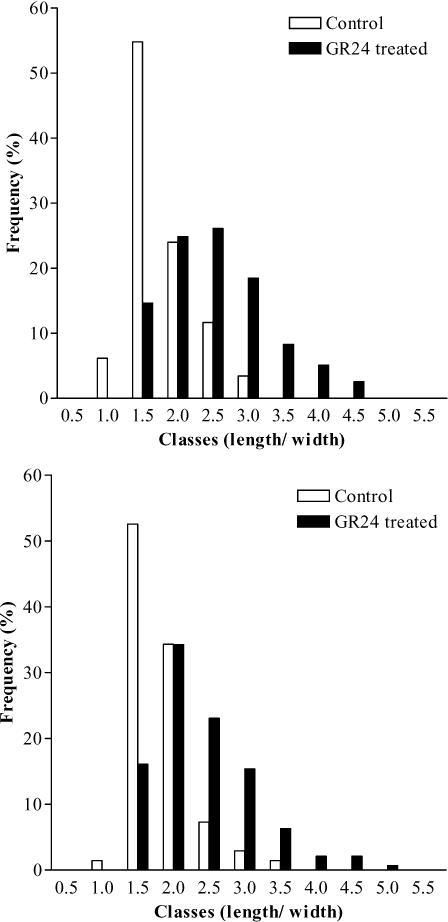
Effect of GR24 on Size of Mitochondria in Hyphae of *Gi. rosea* Stained with Mitotracker Green Hyphae were treated with 30 nM GR24 or with 0.001% acetone for 1 h (A) or 5 h (B). Results are expressed in frequency (%) distribution of length/width ratios ( *n* = 140–150 mitochondria).

Observations of living cells stained with Mitotracker Green showed that the shape and distribution of the organelles was also remarkably modified. In control cells, the mitochondria appeared spherical and randomly distributed, whereas in treated cells they adopted a thread-like structure oriented parallel to the hyphal axis ( Figures 4 and 5). Their motility was also affected: in control hyphae the mitochondria made circular movements and moved short distances, whereas in the GR24-treated hyphae the mitochondria moved faster and in an oriented fashion ( Videos S1– S4). These changes in shape and movement might reflect increased fission and fusion of the mitochondria, typical of active organelle biogenesis [ 23, 24] and consistent with the observed higher mitochondrial density. Taken together, these data show that GR24 provokes a rapid and strong cellular response in *Gi. rosea* that is associated with mitochondrial biogenesis; they suggest that strigolactones are the BF molecules responsible for this response [ 13].

### Strigolactone Analogues GR7 and GR24 Are Active on Phylogenetically Distant AM Fungi

Buée et al. [ 12] reported that the BF of carrot hairy roots was active not only on several *Gigaspora* species but also on the phylogenetically distant AM fungus *Gl. intraradices*. Similarly, Tamasloukht et al. [ 13] verified that the higher mitochondrial activity and rate of respiration seen in *Gi. rosea* in response to carrot BF was also seen in *Gl. intraradices*. The fact that *Gl. intraradices* responds in the same way as *Gigaspora* to BF indicates that the molecular mechanism is widespread among AM fungi. This is important also because the genome of *Gl. intraradices* is currently being sequenced, so this fungus is likely to become the model AM fungus of choice for further investigations.

**Figure 6:**
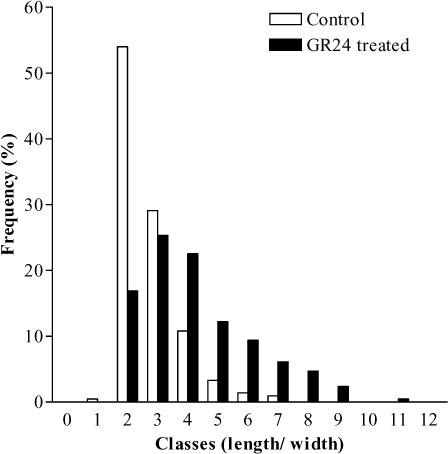
Effect of GR24 on Size of Mitochondria in Hyphae of *Gl. intraradices* Stained with Mitotracker Green Hyphae were treated with 30 nM GR24 or with 0.001% acetone for 1 h. Results are expressed in frequency (%) distribution of length/width ratios ( *n* = 200–220 mitochondria).

The branching response of *Gl. intraradices* is difficult to assess due to its thin, multiple germ tubes; therefore, we replaced this test with a germination test. We observed that 10 ^−7^ M GR7 and 10 ^−7^ M GR24 increased the percentage of spores that germinated by 55% and 50% (300 spores tested per treatment), respectively, compared to their respective controls. We then tested the effects of GR7 and GR24 on the rate of respiration of *Gl. intraradices* spores. *Gl. intraradices* is a suitable species for quantitative measurements of its oxygen consumption in a polarograph due to the small size of its spores and hyphal tubes. We found an average increase of 35% after 5 h in the presence of 10 ^−7^ M GR7 or GR24. This 5-h treatment was too short for the spores to produce new hyphal growth; therefore, the higher rate of respiration in response to GR7 and GR 24 must have been an intrinsic response from pre-existing material rather than a simple cellular growth response. To test the hypothesis that the cellular basis for these physiological responses is mitochondrial as in *Gi. rosea,* we labelled hyphae of *Gl. intraradices* germinated spores with Mitotracker Green. After 1 h of treatment with 30 nM GR24, an increase of 30% fluorescence density was obtained ( *P* < 0.05). Moreover, as in *Gi. rosea,* mitochondria of *Gl. intraradices* appeared more often with a threadlike structure in stimulated hyphae ( Figure 6). Mitochondria of *Gl. intraradices* appeared to move faster than *Gi. rosea* mitochondria, making it impossible to appreciate differences in movement between treated and control mitochondria.

To further validate the status of strigolactones as general AM fungi stimulants, we tested GR7 10 ^−7^ M on a third AM fungal species, *Gl. claroideum*. This species belongs to group B of Glomerales, whereas *Gl. intraradices* belongs to group A [ 25]. Spore germination of *Gl. claroideum* was stimulated in the presence of GR7. The stimulated spores germinated faster, and a higher percent germination was obtained after 4 d of incubation ( Figure S4). These data were validated with a χ ^2^ test (23.78; *P* < 0.05).

## Discussion

We have shown that the BF produced by roots of the monocot *Sorghum* contains a strigolactone and that strigolactone analogues induce strong physiological effects on three diverse AM fungi, as does authentic BF. We have shown also that the BF of the dicot D. carota stimulates seed germination of *Orobanche,* as do strigolactones. Akiyama et al. [ 16] showed that the active ingredient of the BF of *L. japonicus,* another dicot, is a strigolactone. Taken together, these data indicate that strigolactones, previously defined as stimulants of germination of parasitic weeds, are also involved in early AM fungus–host-root interactions in a wide range of plant and fungus species.

The fact that many plant species are hosts to AM fungi [ 26] and/or to parasitic weeds suggests that strigolactones are widespread in the plant kingdom. Yet, due to their very low concentrations and the lack of easy analytical methods to detect them, these small lipophilic molecules have been detected in only a few higher plants [ 27]. Since the discovery of strigol, the first strigolactone to be characterised (in cotton in 1966 [ 28]), only five other natural strigolactones have been described: strigyl acetate, isolated from cotton [ 29]; sorgolactone, from *Sorghum* and maize; alectrol, from cowpea; orobanchol, from red clover [ 15]; and 5-deoxy-strigol, from L. japonicus [ 16]. Recently, an ESI-MS/MS method was developed using multiple reaction monitoring that was suitable for the detection and quantification of several strigolactones in the same sample [ 29]. We also used the ESI-MS/MS but in the enhanced product ion (EPI) mode to be more confident in molecular identification. With this type of analysis, all daughter ions and their respective abundance are visible after collision. This method proved very sensitive in our hands (with a limit of detection of 10 ^−9^ M for standard sorgolactone), enabling us to detect sorgolactone not only in *Sorghum* root exudates but, more directly, in roots and shoots. If strigolactones are also present in shoots of maize, this may explain the presence in maize stem of a non-identified germination stimulant of Striga hermonthica seeds [ 30]. For now, nothing is known about the biosynthesis pathway of strigolactones; however, Matusova et al. [ 31] suggested that strigolactones may be derived from the carotenoid pathway. This hypothesis is consistent with the presence of strigolactones in shoots.

Plants produce thousands of chemicals, many of them with strong biological activities. It would not be surprising, therefore, if AM fungi had learned to interact with many of these chemicals during the course of evolution. Therefore, strigolactones may not be the only one class of molecule in plant roots that stimulates hyphal growth. None of the other stimulants of parasitic weed germination we tested had any activity on *Gi. rosea,* indicating the structural specificity of strigolactones as AM fungal stimulants. Moreover, the very high activity of strigolactones and the fact that they are active not only on *Gigaspora* species: *Gi. margarita* [ 16], *Gi. rosea* (this study), and *Gi. gigantea* (unpublished data) but also on two *Glomus* species (this study) considered to be phylogenetically distant from the Gigasporaceae [ 25], argue in favour of an important role for strigolactones in hyphal growth. Studies with strigolactone mutants or ecotypes of plants that produce less or no strigolactones—ideally in model plants such as Medicago truncatula and L. japonicus—will be crucial to establish whether or not strigolactones are essential for establishing the mycorrhizal symbiosis.

Sorgolactone was active on germinating *Gi. rosea* hyphae at concentrations as low as 10 ^−13^ M, or even lower (considering the dilution effect in the assay; see Materials and Methods). Such high sensitivity has never been reported before in AM symbiosis. The lowest concentrations of flavonoids active in AM fungal growth are in the micromolar range [ 9]. The fact that strigolactones are active at these very low concentrations and that rapid and extensive cellular reorganisation occurs in the fungus suggests that strigolactones act through a signalling pathway. The high turnover of strigolactones in soil [ 32] is also consistent with a signalling role.

The rapid and strong induction of mitochondrial activity in the fungus in response to strigolactones, we believe, illustrates the most significant effect of strigolactone on AM fungi. This mitochondrial response was found in *Gi. rosea* and *Gl. intraradices,* two distant AM fungi. It was correlated to a higher respiration rate with *Gl. intraradices*. We hypothesize that hyphal branching in *Gi. rosea* and activation of spore germination in *Gl. intraradices* and *Gl. claroideum* are physiological consequences of this metabolic stimulation. Activation of the mitochondria leads to oxidation of lipids—the main form of carbon storage in AM fungi. From their observations of fungal spore germination in the absence of roots and in the presence of root exudates, Bécard and Piché [ 33] proposed that a mechanism prevents AM fungal spores from using their carbon reserves until they encounter some specific root exudates. Strigolactones may be that crucial component of root exudates that switches on lipid catabolism in the pre-symbiotic stage of the fungus, which is characterised by the capacity of the fungus to use its full energy potential.

Interestingly, two types of unrelated organisms, AM fungi and parasitic weeds, respond to the same molecules, the strigolactones. These organisms have in common the fact that they are root obligate biotrophs, that their survival relies on efficient host recognition, and that they respond with their germinative structures. The production of strigolactones by plants may well have given them a selective advantage during evolution by encouraging their advantageous association with AM fungi. The parasitic weeds, which appeared much later in evolution than AM fungi, may then have exploited the production of strigolactones by roots for the purpose of colonising their hosts. Thus, the signal transduction systems through which AM fungi and parasitic weeds detect strigolactones may have emerged independently by convergent evolution.

A similar evolutionary mechanism might have given rise, from the ancestral mycorrhizal (Myc) signalling pathway, to the more recent nodulation (Nod) signalling pathway, activated by Nod factors [ 34]. Myc factors have not been isolated and characterised yet, but they are suspected to be essential symbiotic signals produced by AM fungi that activate the plant's symbiotic program. In the legume *M. truncatula,* gene expression and root development respond to (a) diffusible compound(s) produced by AM fungi [ 35– 37]. It will be interesting to investigate whether the strigolactone stimulation of AM fungi is strictly required for Myc factor production, as is the case for Nod factor production by *Rhizobium* in response to plant flavonoids [ 38]. Whether directly or indirectly, we expect that strigolactone stimulation of fungal respiration will increase production of Myc factors, and will help us to isolate and characterise these fungal symbiotic signals.

## Materials and Methods

### Plant materials.

Roots of carrot ( D. carota L.) transformed by the Ri T-DNA of Agrobacterium rhizogenes were routinely cultivated according to Bécard and Fortin [ 39] on minimal (M) medium solidified with 0.4% Gellan gum (Phytagel; Sigma, Steinheim, Germany). The carrot BF was extracted according to Buée et al. [ 12]. Briefly, 500 mg of hairy roots were incubated for 2 d in 100 ml of sterile water in the dark. Crude exudates were filtered and fractionated with ethyl acetate:water (1:1). The lipophilic fraction was dried and re-dissolved in 1 ml of methanol. Insoluble matter was removed by centrifugation, and the methanol phase was diluted with one volume of water. Purification was carried out by HPLC (Spectraphysics, Newport, United States) with a C18 column (Hypurity Elite C18, 250 × 3 × 5 μm, Hypersil, Thermo Electron, Cergy-Pontoise, France). The samples were separated using a gradient from 80:20 (v:v) water/acetonitrile (AcN) to 100% AcN at a constant flow rate of 400μl/min ^−1^, and fractions were collected every 2 min. The absorbancy of the eluates at 300 nm was monitored with a model Spectra 100 detector (Spectraphysics), and the data were integrated with Borwin 1.5 chromatography software (Borwin, Varian, Les Ulis, France). The fraction with activity in the *Gi. rosea* branching assay was obtained in 10:90 water:AcN at 30 min. This fraction was diluted 15-fold in 50:50 methanol:water before use.

Seeds of *Sorghum bicolor* (L.) var. P954063, a variety producing high levels of sorgolactone (provided by D. Hess, Purdue University, West Lafayette, Indiana, United States), were surface-sterilized in 70% ethanol for 30 sec and then in a solution of 10% (w:v) Chloramine T (Sigma, France) for 30 min. After rinsing three times with sterile distilled water, the seeds were transferred aseptically onto 4% potato dextrose agar medium (PDA; Sigma) and allowed to germinate for 4 d at 25 °C in the dark to select the uncontaminated seeds.

Seeds of Orobanche cumana were sterilized in a solution of 4% sodium hypochlorite with 1% Triton X100 for 5 min. Seeds were then rinsed three times in sterile water (5 min per rinse). Two hundred seeds were sown on glass fibre discs (Whatman 9-mm diameter) in glass petri dishes (6-cm diameter) and left to imbibe for 7 d at 21 °C.

### Fungal materials.

Spores of the AM fungus *Gi. rosea* (DAOM 194757) were routinely produced in pot cultures on leeks and collected by wet sieving. They were washed in 0.05% (v:v) Tween 20, soaked with 2% (w:v) Chloramine T (Sigma) for 10 min, washed again three times in sterile water for 30 s per wash, and stored in an antibiotic solution containing 100 mg/l gentamycin and 200 mg/l streptomycin. After 5 d at 4 °C, a second treatment with Chloramine T was carried out under the same conditions. They were then stored at 4 °C before use. *Gl. claroideum spores* (BEG 31) were sterilized twice with the following protocol at 2-d intervals: 30 min in 2% (w:v) Chloramine T at 4 °C, followed by four washes in antibiotic solution (100 mg/l gentamycin and 200 mg/l streptomycin). Sterile spores of *Gl. intraradices* were kindly provided by Premier Tech Ltée (Rivière du Loup, Quebec, Canada).

### Chemicals.

Sesquiterpene lactone solutions of parthenolide and artemisinin (Sigma) were prepared in acetone and ethyl acetate, respectively. They were tested at concentrations from 10 ^−5^ M to 10 ^−9^ M (parthenolide) or from 10 ^−5^ M to 10 ^−13^ M (arteminisin). The hydroquinone series that contains the dihydrosorgoleone at various degrees of unsaturation and resorcinol were extracted from *Sorghum* root tissue according to Erickson et al. [ 40]. Briefly, the roots were dipped in 0.5% HOAc/CH _2_Cl _2_ for 2 s, and the extract was evaporated to give the crude extract *.* To verify that dihydrosorgoleone and resorcinol were the predominant molecules (molecular ion: m/z 360 and 374, respectively) an aliquot dissolved in CH _2_Cl _2_ (100 μl) was analysed by GC-MS on a Hewlett-Packard 5890 gas chromatograph equipped with a 12n HP-1 column and a Hewlett-Packard series 5989A mass detector (electron impact). Chromatography was performed under the following conditions: an initial oven temperature of 60 °C, a ramp of 20 °C/min up to a temperature of 100 °C, and a ramp of 8 °C/min up to a temperature of 290 °C. Total runs lasted 26 min. Resorcinol is believed to be a stabiliser of dihydrosorgoleone. This hydrophobic extract was tested at dilutions from 10 ^−3^ M to 10 ^−7^ M. The strigolactone analogue GR 24 was generously provided by B. Zwanenburg (Nijmegen University, Netherlands) and was prepared in 100% acetone and diluted with water to get 10 ^−7^ M to 10 ^−13^ M final concentrations. GR7 (Argus Fine Chemicals, Brighton, United Kingdom) was diluted 50:50 in methanol:water from 10 ^−5^ M to 10 ^−11^ M. Synthetic sorgolactone was purchased from Chiralix B.V. (Nijmegen, Netherlands). The synthesised product was a mixture of eight diastereoisomers (17% of natural configuration of sorgolactone), and was diluted in methanol from 10 ^−5^ M to 10 ^−15^ M. Activity tests of all these molecules were done using controls systematically prepared with the corresponding solvent.

### Orobanche germination test.

Germination tests of seeds of Orobanche cumana were carried out in the presence of the BF isolated from D. carota hairy root cultures (see above), or in the presence of GR 24 (10 ^−7^ M) as a positive control. After imbibition, 200 μl of each solution to be tested was added to 3 × 200 seeds (200 seeds per glass fibre disk). Negative controls (3 × 200 seeds) were treated with 50% methanol or 0.1% acetone. After 7 d incubation at 24 °C, the number of germinating seeds was counted.

### *Gi. rosea* branching bioassay and growth response.

Branching bioassays were carried out according to Buée et al. [ 12]. Four spores of *Gi. rosea* were germinated (in 2% CO _2_ at 30 °C) on M medium [ 39] supplemented with 10 μM quercetin (Sigma) and solidified with 0.5% Phytagel (Sigma) on each half of two-compartment petri dishes incubated vertically. 5 d after inoculation, each spore produced a single germ tube growing upwards. Two small wells in each half of the petri dish, near the hyphal tip, were cut in the gel with the tip of a Pasteur pipette and 5μl of the solution to test was injected in each well. 24 h later, hyphal branching was recorded either “semi-quantitatively,” based on branch density [ 12], or quantitatively by counting newly formed hyphal branches. The molecules tested were: parthenolide, arteminisin, dihydrosorgoleone (and resorcinol), the strigolactone analogues (GR24, GR7) and synthetic sorgolactone. Hyphal elongation was measured with a 2-mm square grid. Mean values were compared using the one-way Anova test ( *p* < 0.05) and SPSS software (SPSS, Chicago, Illinois, United States). Ten to 20 spores were used for each treatment. The quantitative experiment was repeated three times. A time-course was also carried out with the strigolactone analogue GR7. GR7 was not injected but incorporated, after autoclaving the medium, at a final concentration of 10 ^−7^ M. The number of hyphal branches and the number of centimeters of elongated hyphae (main germ tube plus branches), per germinating spore, were recorded from day 5 to 14. Each experiment was repeated three times with an average of 20 spores per treatment.

### Germination test on *Gl. intraradices*

Solid M medium prepared as above and containing GR7 (10 ^−7^ M) or GR24 (10 ^−7^ M) was poured into 25-well petri dishes. Ten spores of *Gl. intraradices* were added to each well. After 6 d, germination was checked under the stereomicroscope. A total of 300 spores were used per treatment, and the experiment was repeated three times. Variations in germination rates were calculated by comparison with the control germination rate. The significance of values was validated with a one-way Anova test ( *p* < 0.05).

### Germination test on *Gl. claroideum*

Solid M medium containing GR7 (10 ^−7^ M) was prepared as above and poured in small petri dishes (3-cm diameter). Four spores were added per plate. Germination was checked under the stereomicroscope every day after the third day of incubation. A total of 130 spores were used per treatment, and the experiment was repeated twice. The significance of values was validated with a χ ^2^ test ( *p* < 0.05).

### Polarography.

The respiration rate of *Gl. intraradices* in the presence or absence of strigolactone analogues GR7 or GR24 was measured according to Tamasloukht et al. [ 13]. Four hundred spores were germinated in 1 ml of liquid M medium for 6 d in Falcon petri dishes (3-cm diameter). Then, GR7 or GR24 was added to a final concentration of 10 ^−7^ M. Control spores were treated with 50% methanol:water or with 0.1% acetone. After 5 h treatment, the spores were transferred to a Clark electrode inserted into a water-jacketed sealed glass chamber (Hansatech, Norfolk, United Kingdom). The oxygen electrode was connected to a chart recorder calibrated between 0% and 100 % with atmospheric oxygen. Spores were added to the chamber and the temperature was maintained at 30 °C using a circulating water bath. Relative differences of O _2_ consumption were read directly for 15 min from the chart recording. Increased O _2_ consumption in treated spores was calculated by comparison of the slope with the control slope. Three assays and three controls were performed for each experiment. The experiment was repeated three times.

### Cytology.

Mitochondrial densities in *Gi. rosea* and *Gl. intraradices* hyphae were determined after staining mitochondria with the fluorescent probe MitoTracker Green (Molecular Probes, Leiden, Netherlands). After 5–6 d of germination in liquid M medium, germinating spores of either fungal species (ten spores per treatment) were incubated with the dye (1 μM) for 5 h *(Gi. rosea)* or 1 h *(Gl. intraradices)* at 28 °C in the dark under 2% CO _2_. During the staining process, germinated spores were treated with 30 nM GR24 (final concentration in 0.001% v:v acetone), or 0.001% v:v acetone (control) for 1 h *(Gi. rosea* and *Gl. intraradices)* or 5 h *(Gi. rosea).* After treatment, germinated spores were washed with M medium, mounted on glass slides, and observed using an inverted microscope (Leica DMIRBE, Rueil-Malmaison, France) (Excitation, 450–490 nm; Emission, 515nm). Images were acquired with a CCD camera (Color Coolview, Photonic Science, Robertsbridge, United Kingdom) using a 40× immersion oil objective. An average of 35 *(Gi. rosea)* or 65 *(Gl. intraradices)* images of hyphal segments, randomly selected among either treated or control germinated spores, were accumulated in three independent experiments and processed by image analysis (Image Pro Plus, Media Cybernetics, Silver Spring, Maryland, United States). In each hyphal segment the number of bright spots/μm ^2^ was calculated as an estimate of mitochondrial density. Sub-sampled images of treated and control *Gi. rosea* and *Gl. intraradices* hyphal segments were randomly selected for the analysis of the shape of mitochondria. The calculation of mitochondrial length-to-width ratios was made on 140–150 mitochondria for *Gi. rosea* and 200–220 for *Gl. intraradices*, treated and control hyphae. In addition, time-lapse (2 sec) observation of treated and control *Gi. rosea* living hyphae was carried out to observe mitochondrial movements.

For immunolabelling, 20 germinated spores were prepared and treated for 1 h or 5 h with 30 nM GR24 or 0.001% acetone, as above. Hyphae were then fixed with 1% (v:v) paraformaldehyde in water. The fungal cell wall was partially digested with a lytic enzyme mix from Trichoderma harzianum (Acros Organics, Noisy le Grand, France) at a concentration of 12.5 mg/ml in phosphate-buffered saline (PBS, pH 7.4). Non-specific sites were blocked with 0.1% bovine serum albumin (BSA) in PBS (pH 7.4) for 30 min at 28 °C. Hyphae were then incubated for 2 h with mouse anti-yeast monoclonal antibodies against cytochrome oxidase subunit 1 (COX1) (Molecular Probes) or with mouse monoclonal antibodies against yeast porin, diluted in 1% BSA, PBS (pH 7.4) at 1:100 and 1:20 v:v, respectively. After five washes, rabbit anti-mouse antibody coupled to rhodamine or FITC (Molecular Probes) was added diluted 1:50 or 1:100 in PBS (pH 7.4), respectively. Hyphae were incubated for 2 h at 28 °C in the dark with secondary antibodies. Samples were then washed, mounted on glass slides, and observed with an inverted microscope with a 100× immersion oil objective (Excitation 490 nm, Emission 515 nm for FITC; Excitation 515–560 nm, Emission 590 nm for rhodamine). Thirty images of hyphal segments randomly selected among the treated and control germinating spores and labelled with anti-COX1 and anti-porine, in three to five independent experiments, were acquired and processed as above to evaluate mitochondrial density. Statistical analyses of the Mitotracker Green and immunolabelling data were performed using the standard Student *t*-test and SPSS software.

### Sorgolactone production, extraction, and characterisation.

*Sorghum* seeds (around 80) were transferred to a sterilised funnel culture system: a 15-cm diameter filter unit packed with glass wool moistened with 100 ml of sterile distilled water and placed on top of a 500-ml vacuum flask. Each culture unit was incubated in a growth chamber under a day/night cycle of 16/8 h, at 25 °C and 70% relative humidity. At 24-h intervals, root exudates were collected by suction. Every 24 h, the glass wool was washed with 150 ml of sterile distilled water. Root exudate samples were extracted three times with 50 ml of ethyl acetate. The ethyl acetate extracts were dried in a rotary evaporator at 23 °C, redissolved in 2 ml of methanol and stored at −20 °C. They were then pooled and dried in a rotary evaporator, redissolved in 3 ml of water:AcN (70:30, v:v) and chromatographed on a 6-ml C _18_ column Mega Bond Elut (Varian) using increasing concentrations of AcN as the mobile phase: (80:20 water:AcN) fraction 1, (70:30) fraction 2, (60:40) fraction 3, (50:50) fraction 4, (40:60) fraction 5, and (0:100) fraction 6. The fractions were dried in a rotary evaporator and purified by HPLC. 50-μl samples were injected into a 25-cm × 3-mm Hypurity Elite C _18_ reverse-phase column (Hypersil) with a 5-μm pore size. A SP8800 Ternary HPLC Pump (Spectraphysics) was used. The samples were separated using a gradient from 70:30 water: AcN to 100% AcN at a constant flow rate of 400 μl/min, and fractions were collected every 2 min. The absorbance of the eluent was monitored at 239 nm (λ _max_ of synthetic sorgolactone as determined with a UV-Vis spectrophotometer; Cary 100 Scan, Varian). The ESI-MS/MS analyses were performed using a 4000 Q Trap mass spectrometer (Applied Biosystems) with a Turbo V (MDS Sciex, Toronto, Ontario, Canada) source for electro-spray ionisation in the positive ion mode. The mass range of the system is up to 3000 m/z. The samples were infused at a rate of 20 μl/min and monitored in the EPI scan mode at a scan rate of 250 amu/s in the positive ion mode. A standard solution of synthetic sorgolactone at 10 ^−7^ M in AcN:water:HCOOH (90:10:0.1) was used for the optimisation procedure. The nebuliser gas flow was 40 psi, and the capillary voltage was 4400 V. Fragmentation was performed by collision induced dissociation with nitrogen at a collision energy of 28 V. Declustering potential was 90 V. To detect sorgolactone in *Sorghum* tissues, roots and leaves of 4-wk-old *Sorghum* seedlings were ground in liquid nitrogen. Crushed material was extracted, purified, and analysed as above.

## Supporting Information

Figure S1 Molecular Structure of Various Stimulants of Seed Germination of Parasitic Weeds (A) sorgolactone, (B) GR24, (C) GR7, (D) parthenolide, (E) artemisinin, (F) dihydrosorgoleone.
(33 KB DOC)

Figure S2 Immunofluorescence of *Gi. rosea* Hyphae Showing Mitochondrial Protein Staining
Top: Immunolabelling of COX with monoclonal antibody in hyphae treated for 1 h by GR24 3 × 10 ^−8^ M (left), acetone (center), or without primary antibody (right). Secondary antibodies were coupled with rhodamin. Bottom: Immunolabelling of mitochondrial porin with monoclonal antibody in hyphae treated for 1 h by GR24 3 × 10 ^−8^ M (left), acetone (center), or without primary antibody (right). Secondary antibodies were coupled with FITC. Bars = 10 μm.
(43 KB JPG)

Figure S3 Double Immunolabelling of Porin, COX, and Image Overlay Double immunolabelling of porin (FITC, left), COX (rhodamin, middle), and image overlay (right) shows a majority of co-localized, yellow-stained objects. Bar = 10 μm.
(36 KB JPG)

Figure S4 Germination Rate of *Gl. claroideum* Spores on GR7 10 ^−7^ M Medium Compared to Control (50:50 Methanol:Water)
Percent spore germination (130 spores per treatment) was measured every day from day 3 to 13.
(23 KB DOC)

Video S1 Time-Lapse Microscopy Showing Displacement of Mitochondria Stained with Mitotracker Green in Hyphae of *Gi.rosea* Non-Stimulated Control (S1: Apical Region)
In control hyphae mitochondria are spherical and make circular movements, whereas in stimulated hyphae mitochondria have a rod-like shape, and they move faster and in an oriented fashion. Thirty images were acquired per film, and time lapse between two images was of two seconds.
(1.4 MB AVI)

Video S2 Time-Lapse Microscopy Showing Displacement of Mitochondria Stained with Mitotracker Green in Hyphae of *Gi.rosea* Stimulated by GR24 (S2: Apical Region)
(1.3 MB AVI)

Video S3 Time-Lapse Microscopy Showing Displacement of Mitochondria Stained with Mitotracker Green in Hyphae of *Gi.rosea* Non-Stimulated Control (S3: Distal Zones)
(6.7 MB AVI)

Video S4 Time-Lapse Microscopy Showing Displacement of Mitochondria Stained with Mitotracker Green in Hyphae of *Gi.rosea* Stimulated by GR24 (S4: Distal Zones)
(1.9 MB AVI)

## Abbreviations

AM: arbuscular mycorrhizal
BF: branching factor
COX: cytochrome oxidase
EPI: enhanced product ion
HPLC: high-performance liquid chromatography
