# Supplementary material for: Strigolactones Stimulate Arbuscular Mycorrhizal Fungi by Activating Mitochondria
Source: PLoS Biol. 2006 Jun 27;4(7):e226. doi: 10.1371/journal.pbio.0040226 (PMC1481526; doi:10.1371/journal.pbio.0040226)
Supplement: Supplementary file 2 [file pbio.0040226.sg004.doc]

Fig S4
